# *Closo*‐Borate Gel Polymer Electrolyte with Remarkable Electrochemical Stability and a Wide Operating Temperature Window

**DOI:** 10.1002/advs.202106032

**Published:** 2022-04-07

**Authors:** Matthew Green, Katty Kaydanik, Miguel Orozco, Lauren Hanna, Maxwell A. T. Marple, Kimberly Alicia Strange Fessler, Willis B. Jones, Vitalie Stavila, Patrick A. Ward, Joseph A. Teprovich

**Affiliations:** ^1^ Department of Chemistry and Biochemistry California State University Northridge 18111 Nordhoff St. Northridge CA 91330 USA; ^2^ Advanced Manufacturing and Energy Science Savannah River National Laboratory Aiken SC 29803 USA; ^3^ Physical and Life Sciences Directorate Lawrence Livermore National Laboratory Livermore CA 94551 USA; ^4^ Spectroscopy Separations and Material Characterization Savannah River National Laboratory Aiken SC 29803 USA; ^5^ Energy Nanomaterials Sandia National Laboratory Livermore CA 94551 USA

**Keywords:** electrochromic window, gel polymer electrolyte, lithium *closo*‐borate, lithium metal electrode

## Abstract

A major challenge in the pursuit of higher‐energy‐density lithium batteries for carbon‐neutral‐mobility is electrolyte compatibility with a lithium metal electrode. This study demonstrates the robust and stable nature of a *closo*‐borate based gel polymer electrolyte (GPE), which enables outstanding electrochemical stability and capacity retention upon extensive cycling. The GPE developed herein has an ionic conductivity of 7.3 × 10^−4^ S cm^−2^ at room temperature and stability over a wide temperature range from −35 to 80 °C with a high lithium transference number ($t_{\mathrm{Li}}^{+}$ = 0.51). Multinuclear nuclear magnetic resonance and Fourier transform infrared are used to understand the solvation environment and interaction between the GPE components. Density functional theory calculations are leveraged to gain additional insight into the coordination environment and support spectroscopic interpretations. The GPE is also established to be a suitable electrolyte for extended cycling with four different active electrode materials when paired with a lithium metal electrode. The GPE can also be incorporated into a flexible battery that is capable of being cut and still functional. The incorporation of a *closo*‐borate into a gel polymer matrix represents a new direction for enhancing the electrochemical and physical properties of this class of materials.

## Graphical abstract

A lithium *closo*‐borate based gel polymer electrolyte (GPE) demonstrates compatibility with a lithium metal anode and a series of electroactive materials. The GPE delivers high‐capacity retention at high current densities and operates over a wide temperature range from 80 °C down to −35 °C.

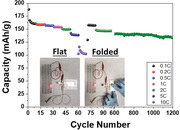

## 1 Introduction

Lithium‐ion batteries (LIB) are a ubiquitous part of daily life from portable electronic devices to the electric vehicles. LIB enabled the mobile electronic revolution owing to it high gravimetric energy density relative to lead‐acid, nickel–cadmium, and nickel metal hydride batteries for this type of application.^[^ ^1^ ^]^ However, high energy density comes with an inherent safety risk if the battery suffers a catastrophic failure and releases all of its stored chemical potential energy at once. In many of these instances, the primary cause of the catastrophic failure of the battery is due to the failure of the electrolyte system. The electrolyte system typically consists of a porous polymer membrane (polypropylene, polyethylene), which is saturated with a lithium salt (lithium hexafluorophosphate (LiPF_6_), lithium perchlorate (LiClO_4_), etc.) dissolved in a liquid carbonate solvent.

The issues associated with traditional electrolytes led to a resurgence in the investigation of solid‐state electrolytes that eliminate the combustible solvent.^[^ ^2^ ^]^ Many superionic oxides,^[^ ^3^ ^]^ sulfides,^[^ ^4^ ^]^ halides,^[^ ^5^ ^]^ polymers,^[^ ^6^ ^]^ and hydrides^[^ ^7^, ^8^ ^]^ have been proposed and employed as solid‐state electrolytes. Of particular interest is the development of polymer electrolytes which can be divided into two broad classes: solid and gel polymer electrolyte (GPE).^[^ ^9^ ^]^ While both types of polymer‐based electrolytes have specific strengths, GPEs are particularly attractive due to their ability to combine the high ionic conductivity, observed in liquid electrolytes, with the advantages of the solid‐state electrolytes (mechanical stability and lithium dendrite suppression).^[^ ^10^ ^]^

Hydrides have recently gained a significant amount of attention as electrolyte materials since the discovery of superionic conductivity in LiBH_4_. These initial studies found when LiBH_4_ was heated past its orthorhombic to hexagonal phase transition at 383 K, it exhibited a significantly enhanced lithium‐ion conductivity.^[^ ^7^ ^]^ Since these reports, numerous efforts have been made to improve the utility of borohydride‐based electrolytes. However, recent research in BH_4_^−^ electrolytes, known as ionic conductive hydrides, has shifted to the *closo*‐borates and their derivatives. *Closo*‐borates are hygroscopic, but not pyrophoric like their MBH_4_ counterparts. Additionally, they can be easily dehydrated/desolvated without decomposition under vacuum and heat making them a much safer option. A number of *closo*‐borates and derivatives (B_12_H_12_^−2^, B_11_H_11_^−2^, B_10_H_10_^−2^, CB_11_H_12_^−1^, CB_9_H_10_^−1^, etc.) have shown exceptional Li and Na ionic conductivities in a wide temperature range.^[^ ^11^, ^12^, ^13^, ^14^, ^15^, ^16^ ^]^ A series of nuclear magnetic resonance (NMR), quasielastic neutron scattering, and molecular dynamics calculations have demonstrated the cation translational mobility is correlated with reorientation rate of the *closo*‐borate consistent with a “paddle‐wheel” mechanism and attributed to their weakly coordinating nature.^[^ ^15^, ^17^ ^]^ This cation translational motion and anion reorientation rate can be tuned via temperature (phase transitions), cation vacancies, the type of atom attached to the boron cage (i.e., H, Cl, F, Br), and *closo‐*borate size.^[^ ^11^, ^18^, ^19^ ^]^ Recent efforts have evaluated the use of mixed *closo*‐borate ionic conductors, incorporating a heteroatom to the boron cage, leading to a further enhancement in ionic conductivity.^[^ ^16^, ^19^, ^20^, ^21^ ^]^ Lithium *closo*‐borate salts have also been investigated for use in traditional liquid carbonate electrolytes and redox shuttles for overcharge protection.^[^ ^22^ ^]^ However, the solubility of hydride *closo*‐borates in carbonates is very low and typically requires partial or full halogenation of the boron cage to achieve concentrations of ≈0.5 m resulting in added cost.

One of primary drawbacks with the use of *closo*‐borate solid‐state electrolytes is typically experiments use 50–100 mg of a *closo*‐borate material to prepare a pellet of a suitable thickness, diameter, and mechanical stability for electrochemical characterization. This is an issue because the mass of the solid‐state electrolyte is typically an order of magnitude or more than the active anode or cathode material in the electrochemical cell and is typically >0.5 mm thick. Lastly, the electrolyte must be incorporated with the active material (typically >25 wt%) to facilitate Li or Na ion transport in the electrode and provide access to all the active material. Recent efforts have been made to lower the percentage of electrolyte added to the electrode composite, but eliminating the electrolyte completely from the process is desirable.^[^ ^23^ ^]^ These three factors significantly reduce the gravimetric and volumetric capacity of the cell as a whole and will hinder their use in practical systems. Due to these drawbacks, there is a need to reduce the quantity and thickness of the *closo*‐borate electrolyte layer in order to eliminate the necessity of electrolyte incorporation into the anode or cathode composite.

Provided these challenges within the field of gel polymer electrolytes and *closo*‐borate cages, this work demonstrates effective incorporation of a *closo*‐borate salt into a GPE to achieve exceptional electrochemical performance and stability while simultaneously reducing the required material to make this technology experimentally feasible. The *closo*‐borate GPE described herein is shown to be compatible with lithium metal electrodes and a series of active electrode materials over an extended cycling period; remarkably, this is achieved without incorporation of the *closo*‐borate into the electrode composite. The approach introduced here is applicable to other *closo‐*borates salts and provides novel insight into the use of lithium salts with large weakly coordinating anions in GPEs.

## 2 Results and Discussion

### 2.1 GPE Preparation

**Figure 1:**
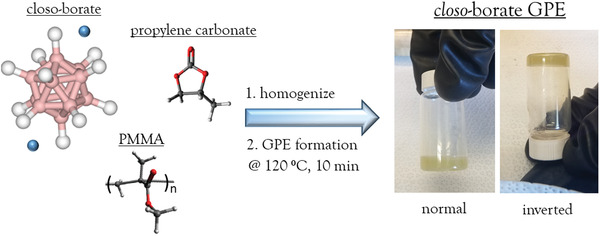
Components of the GPE and photos of the GPE after heating at 120 °C for 10 min and cooling to room temperature.

The preparation of the Li *closo*‐borate infused GPE is described in **Figure** **1**. Once Li *closo*‐borate is dispersed in PC, polymethyl methacrylate (PMMA) is added to the mixture and dispersed with stirring and sonication. The freeflowing solution is heated to 120 °C for 10 min followed by cooling to room temperature to generate the GPE utilized for this study. The *closo*‐borate in the GPE only comprises 5 wt% of the total GPE mass. This is beneficial for reducing the quantity of electrolyte needed per cell and significantly reduces the thickness of the electrolyte layer compared to prior work using bulk powders to prepare electrolytes on the order of 0.5 mm thick. The use of a thin GPE layer will also allow for increase overall cell capacity and ease of manufacture for larger cell formats.

### 2.2 Spectroscopic Characterization

**Figure 2:**
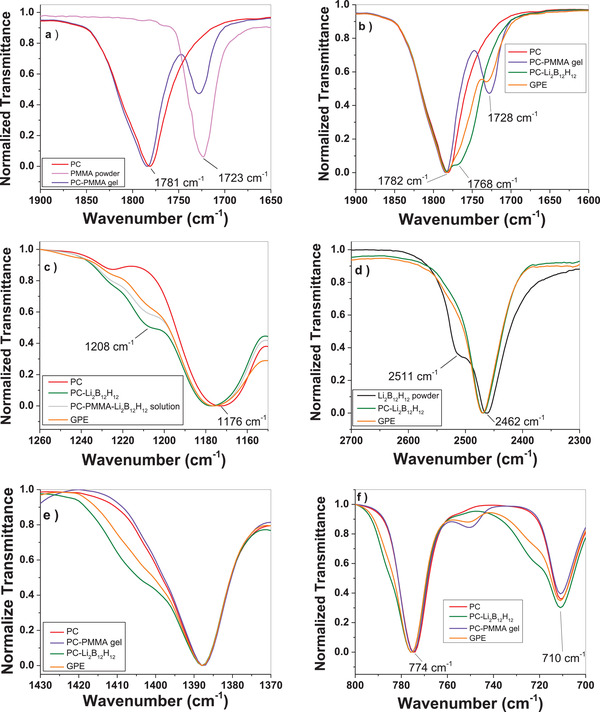
FTIR of the GPE and its components at various stages of the gel formation process. All spectra are normalized to the most intense transmittance band in the region shown. In this study Li_2_B_12_H_12_ powder is black, pure PC is red, PMMA powder is magenta, PC–PMMA gel is violet, PC–Li_2_B_12_H_12_ solution is green, PC–PMMA–Li_2_B_12_H_12_ solution (no gel formation) is gray, and the GPE is orange. a,b) Carbonyl stretching region of PMMA and PC, c) O–C–O stretching mode of PC, d) B–H stretching of B_12_H_12_^−2^, e) —C—H_2_ scissor mode of PC, and f) —C—H_2_ rocking modes of PC.

A series of measurements using various spectroscopic techniques were performed on the components of the gel at various stages of the GPE preparation to understand the chemical environment of each species before and after the gel forming process. First, Fourier transform infrared (FTIR) was used to focus on the carbonyl stretching region of the PMMA and PC as shown in **Figure** **2**a. The spectrum of pure PC liquid and PMMA powder shows carbonyl stretching modes at 1781 and 1723 cm^−1^, respectively. A liquid dispersion of PMMA in PC is nearly identical to pure PC because the solubility of PMMA is negligible as grains of PMMA powder are visible (not shown). When then PMMA–PC mixture is heated to 120 °C for 10 min, the PMMA becomes soluble in PC and forms a viscous translucent gel. The PMMA–PC gel displays a perturbation of the carbonyl stretching modes for both PMMA and PC, by ≈5 cm^−1^ to higher frequency which is attributed the dissolution and solvation of PMMA by PC upon gel formation.

Next, the interaction between the PC and the Li_2_B_12_H_12_ via the carbonyl stretching region of PC was evaluated (Figure 2b). Li_2_B_12_H_12_ is only sparingly soluble in PC and forms a freeflowing liquid when dispersed in PC. The FTIR shows the solvation of the Li^+^ from the carbonyl of the PC as indicated by the formation of a Fermi resonance at 1768 cm^−1^.^[^ ^24^ ^]^ This same behavior has been observed for a number of carbonate based electrolytes with dissolved lithium salts.^[^ ^25^ ^]^ In the GPE, the shoulder attributed to this interaction is still present and noticeable. This Fermi resonance at 1768 cm^−1^ is not observed in the PMMA–PC gel alone. The effect of the Li^+^ solvation from PC is also observable via the stretching mode of the O–C–O portion of the ring structure (Figure 2c). The formation of a new peak at ≈1208 cm^−1^ upon addition of the Li_2_B_12_H_12_ to PC is also consistent with the solvation of Li^+^ with PC.^[^ ^26^, ^27^ ^]^ This previous work also demonstrated that the formation of that peak strictly due to Li^+^ solvation with PC and is independent of the anion pairing.

The solvation structure of the B_12_H_12_^–2^ cage was also analyzed (Figure 2d) via the B–H stretching region for the Li_2_B_12_H_12_ powder, PC–Li_2_B_12_H_12_ solution, and GPE samples. The dry powder shows a split peak at 2462 and 2511 cm^−1^, highlighting a lack of symmetry in the B_12_H_12_^−2^ cage within the crystal structure. Upon addition to PC or in the GPE, the B_12_H_12_^−2^ anion is now in a symmetric environment due to the allowance of free rotation of the B_12_H_12_^−2^ dianion and displacement of the Li^+^ cations from the B_12_H_12_^−2^ as Li_2_B_12_H_12_ becomes solvated by PC. A symmetric solvation environment is also likely to exist around the B_12_H_12_^−2^ anion as indicated by the coalescence of both peaks into one symmetrical peak. This behavior has also been observed for other *closo*‐borates.^[^ ^28^ ^]^ The formation of a symmetrical solvation environment around the B_12_H_12_^−2^, resulting in a single B–H stretching mode for the cage, has also been observed for a Li_2_B_12_H_12_·7NH_3_ complex. In this complex the two Li^+^ are solvated by either three or four ammonia molecules and displaced from the B_12_H_12_^−2^ anion. This indicates the dissolution of the *closo*‐borate salt and formation of solvated and mobile Li^+^. Lastly, the —C—H_2_ scissor mode (1387 cm^−1^) and the —C—H_2_ rocking modes (774 and 710 cm^−1^) present in PC were monitored as shown in Figure 2e,f.^[^ ^29^ ^]^ The scissor mode exhibits a new peak that is formed at 1405 cm^−1^ in a PC–Li_2_B_12_H_12_ solution. This peak is also present, though less intense, in the PC–PMMA–Li_2_B_12_H_12_ liquid and the GPE samples. The rocking modes are also perturbed by the presence of Li_2_B_12_H_12_ with the formation of new peaks formed at 8 cm^−1^ higher in frequency (782 and 718 cm^−1^) relative to pure PC. In the GPE, these two peaks show up as broad shoulders at higher frequencies relative to a pure PC sample. Figure S1a,b of the Supporting Information highlights this shift to higher frequency when a paste of Li_2_B_12_H_12_ in which there is a 1:1 mol ratio of PC:Li_2_B_12_H_12_. Both of these shifts are consistent with the electropositive end of the PC molecule solvating the B_12_H_12_^−2^ and the carbonyl portion of PC solvating Li^+^ because these modes are not affected by the presence of PMMA alone (i.e., PC–PMMA gel). This coordination in the solvation environment is also predicted from density functional theory (DFT) calculations. Raman spectroscopy measurement was performed on the same series of samples (Figure S1c–e, Supporting Information). Perturbations of the vibrational modes observed in the FTIR were also consistent with the Raman spectroscopy measurements.

The local chemical environments of the components were also probed via ^1^H, ^11^B, ^7^Li, and ^13^C NMR spectroscopy and compared to the FTIR analysis. The ^1^H NMR spectra show relatively broad peaks for the Li_2_B_12_H_12_ and PMMA powders as expected for solid‐state samples collected at moderate spinning speeds (Figure S2, Supporting Information). The PC–PMMA gel and GPE ^1^H spectra are dominated by PC chemical environments and display minor contributions from PMMA below 1.5 ppm. The PC–PMMA gel displays the expected chemical shift and splitting patterns for PC liquid along with the PMMA confirmations of the polymer backbone (below 1.4 ppm). The syndiotactic (1.28 ppm), atactic (1.30 ppm), and isotactic (1.33 ppm) conformations of PMMA depend on the arrangement of the pendant methacrylate groups along the backbone of the polymer chain leading to different tertiary structures.^[^ ^30^ ^]^ Upon the addition of Li_2_B_12_H_12_ and formation of the GPE, the signals from these different structures coalesce into one broad resonance at 1.32 ppm and overlap with the protons from the methyl group attached to the PC ring (1.40 ppm) and the protons attached to the B_12_H_12_^−2^ (1.09 ppm) indicating a disordered and fluid polymer backbone. The fluid and disordered nature of the PMMA backbone upon addition of the Li_2_B_12_H_12_ suggests a strong interaction between these components. This indicates that the PMMA polymer is not rigid which creates a flattened energy landscape with multiple energy equivalent sites for Li^+^ to occupy due to its constant motion in the GPE.^[^ ^18a^ ^]^ This results in the Li^+^ not have a preferential site to occupy which facilitates cation hopping between these multiple energy equivalent sites leading to the observed high ionic conductivity (vida infra). The fluid nature and lack of crystalline structure of the PMMA in the GPE was also supported by DSC measurements, since there is no obvious *T*_g_ observed for the PC–PMMA gel or the GPE.

The loss of fine structure for PC protons (3.8–5.0 ppm) and slight shift downfield are expected and consistent with the solvation of Li^+^ and B_12_H_12_^−2^ by PC. The methyl group protons and carbon atoms for PC are slightly shielded and appear shifted upfield in the presence of PMMA compared to pure PC. PC proton and carbon signatures are all appear downfield upon solvation of Li_2_B_12_H_12_. DFT calculations predicted deshielding impacts on all protons and carbons in the PC molecule by coordination with both Li^+^ and B_12_H_12_^2−^ (Tables S2 and S3, Supporting Information). The deshielding of PC protons and carbons persists in the GPE while in the presence of both PMMA and Li_2_B_12_H_12_. Additional pulsed field gradient measurements were performed on the PC–PMMA gel and the GPE. The ^1^H self‐diffusion in the PC:PMMA gel and GPE are 2.06 × 10^−10^ and 6.3 × 10^−11^ m^2^ s^−1^, respectively. The signal from the ^1^H self‐diffusion is primarily attributed to the protons associated with PC. The lower value of the ^1^H self‐diffusion in the GPE relative to the PC–PMMA gel is likely due to the effect of solvation of the Li^+^ and B_12_H_12_^−2^. The presence of these ions in the GPE reduces the amount of “free” (unbound) PC in the system. The mobility of the PC protons associated with solvating the ions will be lower than that of free PC molecules leading to the observed self‐diffusion rates.

^11^B NMR was used to compare the *closo*‐borate powder and the GPE. As expected, a *closo*‐borate mixture was produced based on the closed vessel solid‐state method using LiBH_4_ and B_10_H_14_.^[^ ^31^ ^]^ The *closo*‐borate contains a mixture of lithium hydroborates with Li_2_B_12_H_12_ and Li_2_B_10_H_10_ as the primary species. The primary resonance at −15 ppm is Li_2_B_12_H_12_ with the peaks at −1 and −29 ppm associated with Li_2_B_10_H_10_. Peak fitting indicates that Li_2_B_12_H_12_ is 85% and Li_2_B_10_H_10_ is 12% of the *closo*‐borate powder. Another borate is also present at −16.8 ppm and is 3% of the mixture (Table S1, Supporting Information). This peak could be attributed to the formation of Li_2_B_11_H_11_, LiB_11_H_14_, or possibly other defects in the *closo*‐borate cage structure.^[^ ^32^, ^33^ ^]^ The ^11^B spectra of the GPE have the same peaks as the *closo*‐borate powder although are significantly narrower as a result of the rapid molecular motion of the borate cages. Although this GPE contains a mixture of *closo‐*borates, mixed *closo‐*borate ion conductors have been demonstrate to have higher ionic conductivity than the pure materials.^[^ ^19^, ^20^, ^33^ ^]^ Additionaly, this method demonstrates that using an extremely pure material is not necessary to achieve very favorable electrochemical properties, which can further reduce the cost of the electrolyte.

The ^7^Li NMR of the PC–PMMA–*closo*‐borate liquid (no gel formation) shows only one symmetrical resonance (−0.49 ppm). Upon heating the same solution to form the GPE, a shift of the Li resonance to −0.72 ppm is observed. This corresponds to an increase in shielding observed for Li in the GPE, which suggests additional coordination and electron donation upon gel formation in addition to the PC coordination sphere predicted by DFT calculations. Similar behavior has been observed in other polymers and GPE and is consistent with the interaction of the Li^+^ with the electronegative groups (i.e., C═O) on the PMMA backbone.^[^ ^34^ ^]^ Additional pulse field gradient NMR measurements were preformed to determine the ^7^Li and ^11^B self‐diffusion coefficient (Figure S3, Supporting Information). The values were determined to be 3.63 × 10^−10^ and 3.47 × 10^−10^ m^2^ s^−1^ for ^7^Li and ^11^B, respectively. Utilizing these values and Equation (2), the lithium transference number ($t_{\mathrm{Li}}^{\mathrm{PFG}}$) via the pulsed field gradient NMR method can be extracted (Equation (1)).^[^ ^35^ ^]^ This analysis indicates that the $t_{\mathrm{Li}}^{\mathrm{PFG}}$ is 0.51 for the GPE. This value is significantly greater than that of liquid electrolytes and is attributed to the weakly coordinating nature and large size of B_12_H_12_^−2^ relative to other common anions utilized as electrolytes (i.e., PF_6_^−^). The presence of PMMA could also hinder the mobility of the the large B_12_H_12_^−2^ relative to its mobility in pure PC liquid leading to the observed high transference number and is attibuted to the high viscosity of the GPE. Additionally, the PC molecules that solvate the B_12_H_12_^−2^ will make the effective size of the anion much larger, since it must also carry this solvation sphere to be mobile. This added size will further restict its mobility in the GPE leading to the high transference number

(1) $$t_{\mathrm{Li}}^{\mathrm{PFG}}=\;\frac{D_{\mathrm{Li}}}{D_{\mathrm{Li}}\;+\;D_{B}}$$

**Figure 3:**
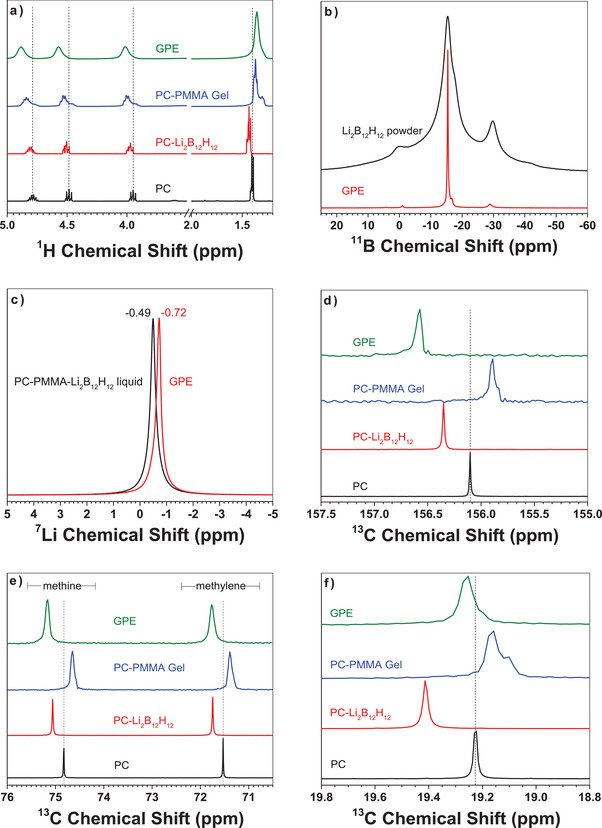
Multinuclear NMR of the GPE and its components. a) ^1^H NMR of a PC–PMMA gel and the GPE, b) ^11^B NMR of the *closo*‐borate powder and the GPE, c) ^7^Li NMR of a PC–PMMA–*closo*‐borate liquid before gel formation and the GPE, d–f) ^13^C NMR of PC, PC–Li_2_B_12_H_12_, PC–PMMA gel, and the GPE focusing on the effect. Dashed lines are to highlight shifts from pure PC.

^13^C NMR spectra in **Figure** **3**d highlight a deshielding of the carbonyl carbon (156.10 ppm) in the presence of Li_2_B_12_H_12_ and in the GPE. This also coroborates a pull in electron density from the carbonyl to the coordinated lithium ion. For the PC–PMMA gel (no *closo*‐borate) a shielding effect is observed with a 0.5 ppm shift to lower ppm for PC carbons. Figure 3e shows the effect on the methine (74.82 ppm) and methylene (71.53 ppm) carbons on the PC ring structure. A similar trend is observed with a deshielding effect observed when the *closo*‐borate is present and a shielding effect when it is not present for all PC carbon shifts. Figure 3f shows the effect on the methyl group (19.22 ppm) attached to the PC ring. A similar trend in shielding/deshielding shifts is observed for this carbon as well. However, the PC–Li_2_B_12_H_12_ now shows the largest deshielding effect while the GPE has the largest deshielding effect for the carbonyl, methine, and methylene carbons of PC. This is attributed to a strong interaction of the electropositive methyl group with the negatively charged *closo*‐borate anion as shown by theoretical calculations. While the GPE does show a slight deshielding shift of this group, the presence of the PMMA attenuates this effect since PC is simultaneous solvating PMMA as well. This is consistent with the shielding shift when a PC–PMMA gel is prepared. The intereaction of PMMA with PC and the *closo*‐borate was also examined by NMR (Figure S4, Supporting Information). Upon gel formation, there is a deshielding effect observed in the ^13^C NMR spectrum (≈1.5 ppm) for the methyl ester group and the methylene carbon of PMMA indicating a relatively strong interaction between the two components.

### 2.3 Theoretical Analysis

**Figure 4:**
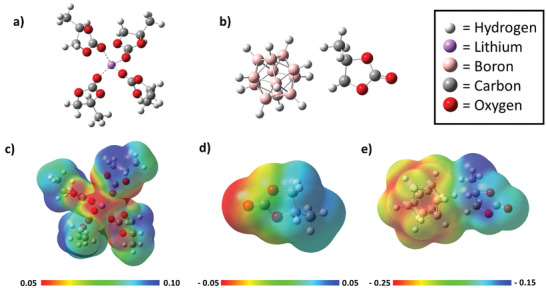
Images of geometry optimized structures from DFT a) Li^+^ solvation with 4 PC molecules, b) optimized structure of the B_12_H_12_^−2^ anion with one PC molecule, c) molecular electrostatic potential surface map of 4 PC molecules coordinating a lithium cation, d) molecular electrostatic potential surface map of PC, and e) molecular electrostatic potential surface map of B_12_H_12_^2−^ dianion coordinated to PC.

First‐principles density function theory (DFT) calculations were performed to gain additional insight into the GPE component interactions. The coordination interactions of three GPE components (PC, Li^+^, and B_12_H_12_^−2^) were calculated to support interpretation of spectroscopic perturbations observed as a consequence of component interactions. **Figure** **4**a illustrates the predicted coordination environment for isolated Li^+^ when solvated by 4 PC molecules. Coordination of Li by 4 carbonate solvent molecules is consistent with predicted coordination environments for other carbonate solvents.^[^ ^27^, ^36^ ^]^ Figure 4b shows the coordination geometry between the B_12_H_12_^2−^ dianion and the PC solvent molecule. Electron density maps, also shown in Figure 4, highlight the perturbation of electron density of the PC molecule upon coordination with the Li^+^ cation and B_12_H_12_^2−^ and a PC molecule. Electron density maps, shown in Figure 4c–e, highlight the perturbation of electron density of the PC molecule upon coordination with the Li^+^ cation and B_12_H_12_^2−^. A substantial redistribution of electron density is observed for PC molecules when coordinated with a lithium cation. The electron density from the 4 PC carbonyls is donated toward the lithium cation. This results in C═O bond elongation for coordinated PC molecules which lowers the energy of the PC carbonyl symmetric stretching mode observed in the FTIR data (1781 cm^−1^) and is consistent with the measured spectra (Figure 2b). The C═O bond lengthens from 1.189 Å predicted for the unbound PC molecule to 1.204 Å when 4 PC molecules are coordinated with a lithium cation. DFT calculations show the electropositive side of the PC molecule is coordinated with the B_12_H_12_^2−^ dianion as expected. This results in a slight redistribution of electron density from the B_12_H_12_^2−^ dianion to the electropositive side of the PC molecule as shown in Figure 4e. In consequence, the C—H_2_ rocking modes (710 and 774 cm^−1^) and C—H_2_ scissor mode (1387 cm^−1^) for PC become blue shifted to higher vibrational energies.

Calculated vibrational frequencies also predict a red shifted C═O stretching mode in PC facilitated by the interactions between the carbonyl group and lithium cation during solvation. The predicted degree of red shift is reduced upon coordination with additional PC molecules due to the electron density pull being more evenly distributed among more C═O groups (Figure S5, Supporting Information). Blue shifts in the vibrational frequencies of the PC —C—H_2_ scissor mode and rocking modes are predicted with coordination to both the lithium cation and B_12_H_12_^2−^ (Figure S6 and Table S4, Supporting Information). Therefore, the higher energy vibrational —C—H_2_ vibrational modes observed for PC in the presence of Li_2_B_12_H_12_ are likely a collective consequence of coordination with both the lithium cation and B_12_H_12_^2−^ dianion. The theoretical predictions carried out on GPE components are consistent with experimental results and prior studies evaluating anion solvation in carbonate electrolytes.^[^ ^37^ ^]^

### 2.4 Electrochemical Characterization

**Figure 5:**
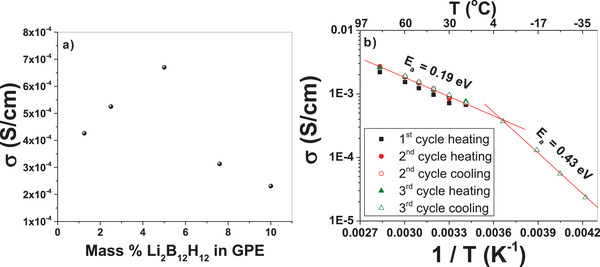
a) Ionic conductivity as a function of mass% Li_2_B_12_H_12_ incoporated into the GPE. b) Ionic conductivity of the GPE obtained from EIS experiments using two stainless steel blocking electrodes from −35 to 80 °C.

The ionic conductivity of the GPE as a function of mass% Li_2_B_12_H_12_ is shown in **Figure** **5**a as measured by electrochemical impedance spectroscopy (EIS) between two polished stainless steel (SS) blocking electrodes. The highest ionic conductivity was achieved with 5 mass% of Li_2_B_12_H_12_ in the GPE. Figure 5b shows the temperature dependent ionic conductivity of the 5 mass% Li_2_B1_2_H_12_ GPE. The ionic conductivity is 6.7 × 10^−4^ S cm^−1^ at 20 °C and consistently increases as the temperature is increased up to 80 °C (2.7 × 10^−3^ S cm^−1^). Remarkably, the ionic conductivity at 20 °C is more than double that of a pure solid‐state Li_2_B_12_H_12_ pellet at the same temperature (3.1 × 10^−4^ S cm^−1^).^[^ ^13^ ^]^ After cooling down to room temperature and measuring the ionic conductivity as a function of temperature again, there is a slight increase in the ionic conductivity when compared to the first heating cycle. Subsequent heating and cooling cycles of the GPE showed consistent ionic conductivity values as a function of temperature after the first initial heating cycle. The ionic conductivity stabilized at an average of 7.3 (± 0.2) × 10^−4^ S cm^−1^ for six different measurements after multiple heating and cooling cycles. This is attributed to better contact achieved at the stainless steel/GPE interface upon heating. The activation energy for Li^+^ conduction through the GPE above 0 °C is 0.19 eV. The ionic conductivity was also measured below room temperature, and the GPE still shows an impressive 1.5 × 10^−5^ S cm^−1^ at −35 °C. The change in slope below 0 °C is likely due to a reduction in viscosity and available degrees of motion in the PMMA/PC gel as the temperature approached the freezing point of PC (−48 °C) and has been observed in other GPEs at low temperature.^[^ ^38^ ^]^ A corresponding increase in the activation energy was also observed for Li^+^ conduction to 0.43 eV in the GPE below 0 °C. This activation energy in the low temperature regime is the same as previously reported for Li_2_B_12_H_12_ in the solid state. This could indicate that the Li_2_B_12_H_12_ salt starts to crystallize and precipitate in the GPE leading to an unsolvated material that behaves similar to bulk Li_2_B_12_H_12_ powder at lower temperatures.

This high values of the transferrence number and ionic conductivity is attributed to the combined effect of the weakly coordinating nature, delocalized negative charge, and the relatively large size of the B_12_H_12_^−2^. The combination of these factors contributes to these properties. One factor that could lead to enhanced ionic conductivity in the proposed GPE, is the low lattice energy (Δ*H*_latt_) of the lithium *closo*‐borate salt based on theoretical Born–Haber cycle calculations.^[^ ^39^ ^]^ For example, Li_2_B_12_H_12_ has a lower than expected lattice energy and indicates that the attraction between the B_12_H_12_^−2^ and the two Li^+^ cations is low. The low affinity (or weakly coordinating nature) of *closo*‐borates for cations has also been confirmed experimentally.^[^ ^12^, ^40^ ^]^ A low affinity of the B_12_H_12_^−2^ will facilitate the dissociation of the cations when incorporated into the GPE. The low affinity between the two will also minimize the likelihood of ion pairing. Ion pairing occurs when the anion recombines with the cation in the electrolyte system, which eventually forms triplets and high aggregates. Ion pairing decreases the ionic conductivity and the lithium ion transference number while increasing polarization losses in batteries due to a reduction in the free charge carriers present in the system.^[^ ^41^ ^]^ Larger anions will also exhibit a higher level of negative charge delocalization when compared to smaller anions. This large negative charge delocalization is also expected to reduce ion pairing in the GPE. The use of large anion salts is further supported by recent work that developed a PEO‐based polymer electrolyte infused with a lithium salt containing a carbon quantum dot.^[^ ^42^ ^]^ They were able to achieve high ionic conductivity (2.02 × 10^−4^ S cm^−1^) and a transference number of 0.99. The use large anion containing lithium salts represents a direct and straightforward approach to enhance the electrochemical properties of electrolytes because the size and distribution of surface charge of the anion can be fine‐tuned and optimized for the operating conditions.

The frequency dependence of the dielectric permittivity or dielectric constant (*ɛ*′) for the GPE is shown in Figure S7a of the Supporting Information. The *ɛ*′ defines the ability of a material to become polarized upon application of an electric field. At low frequency, the ions in the GPE can readily realign with the direction of the oscillating electric field, which results in an accumulation of ions at either electrode (polarization). This results in a high dielectric constant and indicates the dissociation of the Li_2_B_12_H_12_ salt to form more charge carriers in the GPE. As the frequency of the oscillating electric field is increased, the ions are unable to respond and do not accumulate at the electrodes resulting in a lower value for the dielectric constant. As the temperature of the GPE is increased from −35 to 80 °C, the dielectric constant also increases and is due to increased vibrational and translational energy of the GPE components. The dielectric loss (*ɛ*″), which is a measure of the energy required to align the dipoles in the direction of the field (Figure S7b, Supporting Information). A similar trend is observed for *ɛ*″ with high values at low frequency and low values at higher frequencies and is consistent with the frequency dependent *ɛ*′ data. The increase in temperature also reduces the interaction between the ions facilitating further dissociation and increase in charge carriers. The loss tangent (tan *δ*) was also calculated and is the ratio of the mobile and stored dipoles in the material (Figure S7c, Supporting Information). The tan *δ* shows a single relaxation peak, which indicates ionic conduction in the present system. The relaxation peak shifts to higher frequency as a function of temperature. At the peak maximum, the frequency of molecule rotation matches the frequency of the applied electric field. This resonance leads to the maximum power transfer to the dipoles in the system resulting in the maximum heat loss at this frequency. This finding is consistent with the increase in conductivity as a function of temperature due to the increase of mobile charge carriers in the system.

**Figure 6:**
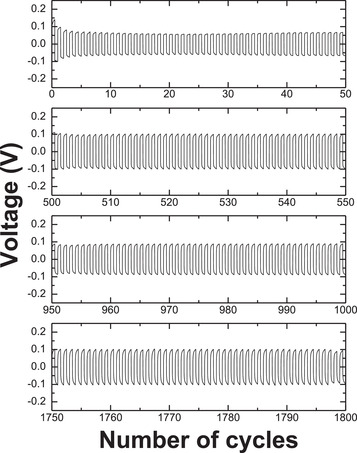
Long‐term constant current cycling of a symmetrical cell (Li/GPE/Li) at ±0.25 mA cm^−2^ operated at 20 °C. The duration of each cycle was 1 h.

The cyclic voltammogram (Figure S8, Supporting Information) demonstrates the reversible stripping and plating of lithium on an SS electrode below 0 V relative to lithium metal for 3 cycles. Constant current cyclic measurements of a symetrical Li/GPE/Li at room temperature is shown in **Figure** **6**. The GPE shows excellent stability and compatibility with the metallic lithium electrode for over 1800 cycles at room temperature. In this experiment, constant current was applied in the forward direction for 30 min, then in the reverse direction for 30 min to complete one full cycle.

**Figure 7:**
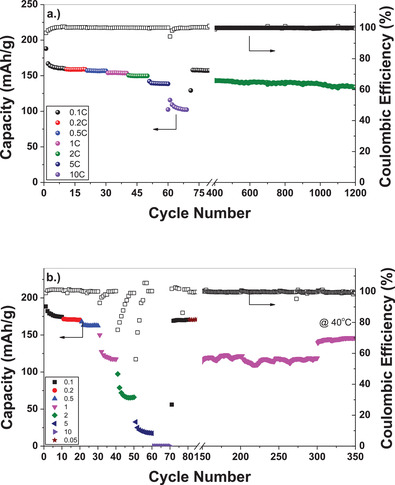
a) Galvanostatic cycling of the LTO anode with the GPE at various rates. b) Galvanostatic cycling of the TiS_2_ cathode with the GPE at various rates.

To determine if the Li_2_B_12_H_12_ based GPE is viable as an electrolyte, it was first tested in LTO and TiS_2_ half cells (**Figure** **7**a,b). In this half‐cell setup, the electrodes were cast onto metal foil current collectors from a slurry, which contained 80 wt% active material (LTO, TiS_2_), 10 wt% acetylene black, and 10 wt% polyvinylidene fluoride (PVDF) polymer binder. No *closo*‐borate is incorporated into the electrode composite. The LTO half‐cell demonstrated excellent performance and cycle retention even at high current densities (10 C; 6 min charge/discharge). Substantial capacity retention was observed for over 1200 cycles at 2 C (30 min charge/discharge). The TiS_2_ cathode also demonstrated extended cycle stability with reasonable capacity retention for 350 cycles. However, a significant reduction in capacity was observed at current densities greater than 1 C. The extended cycling at 1 C (cycles 85–300) showed periodic oscillations, which is attributed to slight changes in the temperature of the room over the course of a day. This oscillation is less noticeable for the last 50 cycles (cycles 300–350) in which the cell was heated to 40 °C for cycling. The increase in capacity is expected and attributed to the higher ionic conductivity of the GPE at that temperature. The GPE still has a much lower ionic conductivity at −35 °C than at room temperature (7.3 × 10^−4^ S cm^−1^ vs 1.5 × 10^−5^ S cm^−1^). However, it was demonstrated that 3 LEDs can still be powered utilizing a Li/GPE/TiS_2_ half‐cell at −35 °C (Figure S9 and Video S1, Supporting Information).

**Figure 8:**
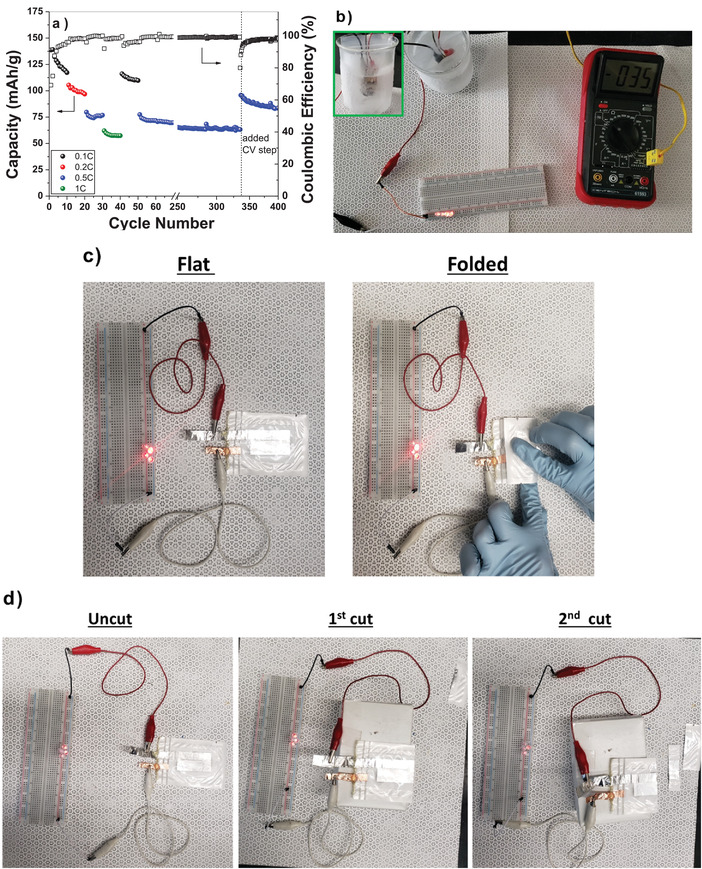
a) Cycling study of the LiFePO_4_ with the GPE. The added constant voltage (CV) step to the charging segment after the 335th cycle is indicated on the plot. b) Screen shot from Video S2 of the Supporting Information demonstrating the ability of the Li/GPE/LFP cell to power 4 LEDs at −35 °C. Inset in the top left of the image is close‐up of the coin cell in MeOH‐dry ice slush bath (Video S2, Supporting Information). c) Images of a flexible cell with the GPE and a LiFePO_4_ cathode lighting 4 LEDs in the flat and folded states (Video S3, Supporting Information). d) Images of a flexible cell with the GPE and a LiFePO_4_ cathode lighting 4 LEDs intially uncut and after two cuts (Videos S4 and S5, Supporting Information).

The cycle stability of the GPE with a LiFePO_4_ cathode is shown in **Figure** **8**a. Although there is some initial capacity loss with the first few cycles, the capacity shows improved stability with extended cycling. Capacity stability is demonstrated for long term cycling at 0.5 C for 350 cycles. The first 350 cycles were performed at the indicated charge/discharge rates. After the 335th cycle, a constant voltage step (hold at 3.8 V for 1 h) was added to the charging segment of the cycle resulting in the observed capacity increase. The added constant voltage step allowed additional time for the extraction of lithium from a larger portion of the LiFePO_4_ cathode. Upon returning to 0.1 C after the 400th cycle, the capacity is 84 mAh g^−1^ showing an excellent capacity retention. Figure 8b is an image taken from Video S2 of the Supporting Information, which shows that the Li/GPE/LFP cell can light up 4 LEDs at −35 °C after equilibrating at this temperature for >20 min. A flexible LiFePO_4_ battery using the GPE was also successfully fabricated (Figure 8c; Video S3, Supporting Information) and tested. The flexible battery was able to continuously light LEDs during multiple bending cycles. After the bending cycles, the same cell was tested to determine if it was capable to still light the LEDs after being cut twice (Figure 8d; Videos S4 and S5, Supporting Information). The flexible cell, even when cut twice, still showed the ability to power the LEDs, which further enhances the potential use of this GPE in conformal and safer batteries. The ability to stay lit, even under these conditions, is attributed to the sticky nature of the GPE which holds good contact with the electrodes and allows for the continued flow of Li^+^ between the two.

The GPE was also demonstrated to be compatible with the organic cathode active material perylenetetracarboxylicdiimide (PTCDI), which has a relatively flat discharge plateau at 2.4 V versus Li metal (Figure S10, Supporting Information).^[^ ^43^ ^]^ Organic cathodes are of interest due to their potential as sustainable replacements for traditional transitional metal oxide based cathodes.^[^ ^44^ ^]^ The PTCDI cathode was paired with a Li metal anode using the GPE and cycled at 50 mA g^−1^. The cell shows a reversible capacity of 98 mAh g^−1^ after 300 charge/discharge cycles.

**Figure 9:**
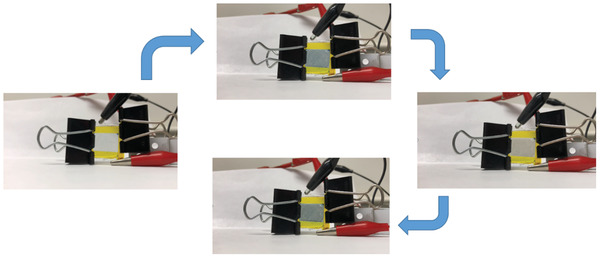
Screenshots demonstrating the use of the GPE as the electrolyte for a Prussian blue‐based electrochemical window (Video S6, Supporting Information).

Next, the GPE was used as the electrolyte for an electrochromic window (**Figure** **9**) based on Prussian blue (PB) and zinc hexacyanoferrate (ZnHCF) nanocubes as the electrochromic material and the ion storage layer cast on an ITO/glass substrate, respectively.^[^ ^45^ ^]^ The PB and ZnHCF were first synthesized and suspended in an aqueous solution that was spin‐coated onto the ITO/glass substrate. The GPE was then sandwiched between the two electrodes with Kapton tape as a spacer inside of a glovebox. Upon lithiation of the Prussian blue electrode, the reduction of Fe^III^ to Fe^II^ produces Prussian white, which is colorless (Equation (2)). The ZnHCF is colorless in the lithiated and delithiated states (Equation (3)). The reversible color switching is achieved by applying a + or −2 V potential (Video S6, Supporting Information)

(2) $${\mathrm{Fe}}_{4}^{\mathrm{III}}{\left[Fe^{\mathrm{II}}{\left(\mathrm{CN}\right)}_{6}\right]}_{3}+4Li^{+}+4\;e^{-}\;↔Li_{4}{\mathrm{Fe}}_{4}^{\mathrm{II}}{\left[Fe^{\mathrm{II}}{\left(\mathrm{CN}\right)}_{6}\right]}_{3}$$

(3) $$2Li_{2}{\mathrm{Zn}}_{3}^{\mathrm{II}}{\left[Fe^{\mathrm{II}}{\left(\mathrm{CN}\right)}_{6}\right]}_{2}\;↔2{\mathrm{Zn}}_{3}^{\mathrm{II}}{\left[Fe^{\mathrm{III}}{\left(\mathrm{CN}\right)}_{6}\right]}_{2}\;+4Li^{+}+4\;e^{-}$$

## 3 Conclusion

In this work, incorporating a lithium *closo*‐borate salt into a PC–PMMA gel to form an extremely stable GPE that has a wide temperature operating window (−35 to 80 °C) was demonstrated. The high ionic conductivity of the GPE is attributed to the weakly coordinating nature of the large *closo*‐borate anion and relatively low affinity for Li^+^, which facilitates high ionic conductivity. The GPE only contained 5 wt% of the *closo*‐borate and is more than an order of magnitude thinner than solid‐state cells that have previously utilized a *closo*‐borate electrolyte. A series of spectroscopic measurements and DFT calculations evaluated the interaction between the components of the GPE to obtain chemical information about the unique local environments of Li, B, C, and H. Compatibility between the GPE and lithium metal in symmetrical cells along with LTO, TiS_2_, LiFePO_4_, and PTCDI electroactive materials was confirmed. A flexible cell that was capable of being cut can still safely power LEDs was also demonstrated. Finally, GPE was utilized as an electrolyte in an electrochromic device based on the Fe^III^/Fe^II^ redox couple of Prussian blue. The approach used in this study to enhance the electrochemical and physical properties of *closo*‐borate electrolytes represents a new direction of research for electrolytes based on weakly coordinating polyborate anions. The rich chemistry of *closo*‐borates, variety of plasticizers, tunability of polymers, and nanodomain additives currently used in other GPE systems indicate that significant improvements are readily achievable. The findings suggest this approach can lead to further development and advancement of other similar borate salts in a variety of energy storage and conversion technologies that will be reported in due course.

## 4 Experimental Section

### Materials

Lithium borohydride (LiBH_4_) and PMMA ≈350 000 MW were purchased from Sigma‐Aldrich. Acetylene black (AB) and decaborane (B_10_H_14_) were purchased from Alfa Aesar. PC was purchased from Acros Organics. PVDF was purchased from MTI Corp.

### Li_2_B_12_H_12_ Synthesis and GPE Preparation

Lithium dodecahydro‐*closo*‐dodecaborate, Li_2_B_12_H_12_ (referred to as lithium *closo*‐borate) was prepared in an argon filled glovebox following a procedure previously described.^[^ ^31^ ^]^ Briefly, stoichiometric ratios of lithium borohydride, LiBH_4_, and decaborane, B_10_H_14_, were measured into a stainless steel ball mill and sealed. These materials were milled together using an MSK‐SFM‐Desk‐Top High Speed Vibrating ball mill with 50 g of stainless steel balls for 45 min. The ball milling was done in 5 min intervals. Each 5 min of ball milling was followed by a 5 min cool down period. After every 10 min of ball milling, the material was brought back into the argon glovebox to be scraped down to encourage even milling. The resulting material was then measured into a stainless steel Swagelok cell, sealed using copper gaskets, and annealed at 200 °C for 18 h in a CF1100 Muffle Furnace. The Swagelok cell was then brought back into the argon glovebox and the annealed material was collected and ground using a mortar and pestle. The resulting Li_2_B_12_H_12_ powder is light yellow in color. For the LiFePO_4_ cycling data, a pure sample of Li_2_B_12_H_12_ was utilized rather than the mixture of *closo*‐borates that result from the previously described solid‐state synthesis. The pure Li_2_B_12_H_12_ powder sample was prepared from Cs_2_B_12_H_12_. First, the Cs_2_B_12_H_12_ was dissolved in water then passed through a cation exchange resin (Amberlite IRC120, hydrogen form, Sigma) to obtain the acid form of B_12_H_12_^−2^. An aqueous LiOH solution was added to the *closo*‐borate solution until a neutral pH was obtained. The water was then removed by rotary evaporation to obtain a white powder. The powder was then dried under vacuum at 250 °C for at least 5 h before use.

The polymer electrolyte was prepared in a 20 mL borosilicate glass vial. Li_2_B_12_H_12_ powder (0.18 g) was very slowly incorporated into anhydrous propylene carbonate (3.0 g) with stirring and evenly dispersed via sonication to mitigate the formation of large agglomerations of material. The polymer, polymethyl methacrylate (0.80 g), was then added to the plasticizer and *closo*‐borate salt solution. The formation of the gel was induced by heating the PC, PMMA, and Li_2_B_12_H_12_ mixture to 120 °C for 15 min. This results in ≈0.5 m Li_2_B_12_H_12_ semiopaque light‐yellow gel, further referred to as the GPE.

### Anode/Cathode and Coin Cell Preparation

Cathode materials were prepared on aluminum foil while anode materials were prepared on copper foil. A slurry was made using the following basic formula: active material (70–80 wt%), conductive carbon (10–2.5 wt%), and a binder (10–2.5 wt%). The active materials included LTO for anode preparations and TiS_2_, PTCDI, and LiFePO_4_ for cathode preparations. The conductive carbon utilized was AB and the binder utilized was PVDF. The respective amounts of each powder were measured and homogenized using a mortar and pestle. A slurry was prepared by adding minimal amounts of n‐methyl‐2‐pyrrolidone, with constant stirring. The prepared slurry was allowed to mix and pipetted onto the respective current collector and cast into a 11 µm thin film using a micrometer doctor blade. The thin film was allowed to dry overnight at 80 °C. TiS_2_ active material slurries were sustained in an inert environment throughout preparation and cast in an argon glove bag. Once dry, 10 mm disks of the material were cut out using an individual hole punch. The loading density of the active material for the electrode tested was 1.0 mg cm^−2^. The flexible cell consisted of a LiFePO_4_ cathode, GPE, and Li metal anode that was sealed inside of a Mylar bag. Copper and aluminum tape were utilized as the anode and cathode tabs.

Coin cells were prepared using CR 2032 cases. A 10 mm disk of Li foil was cut and placed at the center of the coin cell base. Using a pipette, a small amount of the heated Li_2_B_12_H_12_ GPE was applied to the lithium and layered with an 18 mm disk of glass fiber filter paper to act as a separator. Another small amount of the GPE was then layered on top of the glass fiber filter paper. The anode or cathode disk was applied with the active material side down onto the GPE. To ensure good contact of the electrodes with the GPE, a stainless steel disk and spring were centered on top. The coin cell was closed using the cap and the cell was sealed using a Digital Pressure Controlled Electric Crimper. All electrochemical experiments were performed on BioLogic VMP3 multichannel potentiostat.

The electrochromic window was prepared utilizing Prussian blue and white analogues as previously described for other electrolyte systems.^[^ ^45^ ^]^ The aqueous suspension of these two components was spin coated onto separate, cleaned ITO on glass substrate. The coated substrates were then allowed to dry before transferring them into the glovebox for device assembly. The GPE was applied to the coated substrate using Kapton tape as a spacer between the two substrates and held together with clips before removing from the glovebox. A potential of + or −2 V was applied to achieve the electrochromic properties.

### FTIR and Raman Spectroscopy

Raman spectroscopy measurements were carried out by irradiating samples with a 785 nm laser (Laser Process Instruments). The beam passed through a 785 nm laser line filter and was focused down into sample vials (containing samples under argon) using a 60 mm focal length lens. Raman scattered light from the samples passed through a 785 nm long pass filter and was directed to an optical fiber leading to a spectrometer using a 35 mm focal length lens. The detection system was an iHR320 spectrometer (Horiba). The spectrometer is equipped with three selectable gratings of various groove densities. A custom software suite (SRNL) was used to define the spectrometer parameters and collect spectra. The spectrometer was wavelength calibrated using Ne, Ar, and Kr pen‐ray lamps (Ultra‐Violet Products). An exposure time of 30 s with three iterations was used for each spectrum. Spectra were collected using a 1200 groove mm^−1^ grating at a spectral range centered at 820 nm, which was increased in 40 nm increments up to a final spectral range centered at 1060 nm. FTIR was performed on a Perkin‐Elmer Spectrum Two with a diamond Universal ATR accessory. The samples were transferred from the glovebox to the instrument and analyzed under inert atmosphere utilizing a homebuilt enclosure to prevent reaction with air and moisture.

### DFT Calculations

Quantum mechanical calculations were conducted using the Gaussian 16 software platform.^[^ ^46^ ^]^ The initial geometry of B_12_H_12_^−2^ was optimized using the hybrid B3LYP functional and Pople 6–311g triple zeta basis set with polarization and diffuse functions on all atoms. Explicit solvation interactions were calculated using the same functional and basis set by adding propylene carbonates sequentially using the previous geometry optimization as a starting point. Vibrational frequency and nuclear magnetic resonance calculations were carried out on geometry optimized structures to provide insight into the nature of solvent/analyte interactions and to support experimentally obtained spectra of the electrolyte materials. Structures were rendered in the GaussView 6 software platform.^[^ ^47^ ^]^

### Multinuclear NMR

Measurements were collected at Larmor frequencies of 100.60, 128.37, 155.49, and 400.09 MHz for ^13^C, ^11^B, ^7^Li, and ^1^H, respectively. The samples were packed in an Ar glovebox within 4 mm ZrO_2_ rotors with O‐ring caps and spun at 2 kHz for the gel samples and 10 kHz for the solid samples. For observing the quadrupolar nuclei, ^7^Li and ^11^B, hard RF pulses (*π*/18) of length 0.5 µs were used for obtaining semiquantitative spectra and uniform excitation of the entire spectral region. Both experiments were performed with a recycle delay of 2 s and using 1H decoupling during acquisition with spinal64. ^13^C spectra were collected with a *π*/2 pulse (5.5 µs) with a 5 s recycle delay and tppm 1H decoupling. ^1^H spectra were collected using a *π*/2 pulse (3.625 µs) and with a 3 s recycle delay. Self‐diffusion coefficients of ^1^H, ^7^Li, and ^11^B were measured using a pulse field gradient (PFG) NMR sequence on a Doty Scientific 4 mm magic angle gradient probe. The GPE sample was loaded into a 40 µL Kel‐F sealing cell and capped with a Teflon plug to constrain the gel during measurements and spun at 3.3 kHz for the PFG measurements. Diffusion coefficients were collected using a stimulated echo sequence with bipolar half sine gradient pulses with an eddy current delay before detection and spoiler gradients to remove undesired coherences. The sample temperature was determined indirectly with an NMR thermometer by measuring the ^207^Pb chemical shift of lead nitrate while performing a diffusion measurement under identical conditions (spinning speed, gradient pulse strength and length) and was found to be 24.9 °C throughout the duration of the diffusion measurement.^[^ ^48^ ^]^ Signal attenuation was recorded with 16 increments as a function of increasing gradient strength g from 0.043 Tm^−1^ up to 2.14 Tm^−1^ and fit to the Stejskal–Tanner equation to determine the diffusion coefficient^[^ ^49^ ^]^

(4) $$S\;=\;S\left(0\right)\exp\left[-D{\left(\frac{4\delta g\gamma }{\pi }\right)}^{2}\left(\Delta -\frac{\tau }{2}-\frac{2\delta }{3}-p_{\pi }\right)\right]$$

where *D* is the self‐diffusion coefficient, *γ* is the gyromagnetic ratio, *δ* is the gradient pulse length, *g* is the gradient strength, Δ is the diffusion time, *τ* is the gradient recovery time, and *p*_*π*_ is the *π* pulse length. The ^1^H PFG experiment was collected with a *π*/2 pulse = 5.625 µs and *π* pulse = 11.25 µs (RF field strength ≈44 kHz) and gradient pulse length *δ* = 2.5 ms, diffusion time Δ = 24 ms, gradient recovery time *τ* = 0.2 ms and eddy current delay was 6 ms. The RF pulse parameters for ^7^Li and ^11^B PFG MAS measurements used a *π*/2 pulse length of 10 µs (RF field strength ≈25 kHz) and *π* pulse of 20 µs. Both ^7^Li and ^11^B PFG were collected with the same gradient pulse parameters of gradient length *δ* = 4 ms, diffusion time Δ = 6 ms, gradient recovery time *τ* = 0.2 ms, and eddy current delay was 1.5 ms. ^7^Li, ^13^C, ^11^B, and ^1^H chemical shifts were externally referenced to 1 m LiCl aq (*δ*_iso_ = 0 ppm), the methylene peak of adamantane (*δ*iso = 38.48 ppm), boric acid (0.1 m H_3_BO_3(aq)_, *δ*_iso_ = 19.6 ppm), and the ^1^H resonance of adamantane (*δ*_iso_ = 1.75 ppm), respectively. Solution state NMR was collected on selected samples using a Bruker Avance III 400 NMR.

### Statistics

The PFG NMR measurements were collected on a single sample loaded into a liquid sealing cell at 24.9 °C. The data were fit to the Stejskal–Tanner equation (Equation (4)) and produced an R‐squared value of 0.99087 for the ^1^H data, 0.91084 for the ^7^Li data, and 0.96827 for the ^11^B data. Ionic conductivity was measured on three different individually prepared GPE samples and measured at least three times at each indicated temperature. The standard deviation (s) was calculated via $=\sqrt{\frac{\sum (x_{i}-\bar{x})}{n-1}}\;$, in which *x_i_* is the value of an individual trial, $\bar{x}$ is the average of the trials, and *n* is the number of trials in the data set.

## Conflict of Interest

The authors declare no conflict of interest.

## Supporting information

Supporting Information

Supplemental Video 1

Supplemental Video 2

Supplemental Video 3

Supplemental Video 4

Supplemental Video 5

Supplemental Video 6

## Data Availability

The data that support the findings of this study are available from the corresponding author upon reasonable request.
