# Supplementary material for: Closo‐Borate Gel Polymer Electrolyte with Remarkable Electrochemical Stability and a Wide Operating Temperature Window
Source: Adv Sci (Weinh). 2022 Apr 7;9(16):2106032. doi: 10.1002/advs.202106032 (PMC9165492; doi:10.1002/advs.202106032)
Supplement: Supplementary file 1 — Supporting Information [file ADVS-9-2106032-s007.pdf]

Supporting Information

Closo-borate gel polymer electrolyte with remarkable electrochemical stability and a wide operating temperature window

Matthew Green, Katty Kaydanik, Miguel Orozco, Lauren Hanna, Maxwell A.T. Marple, K. Alicia Strange Fessler, Willis B. Jones, Vitalie Stavila, Patrick A. Ward, Joseph A. Teprovich Jr.**

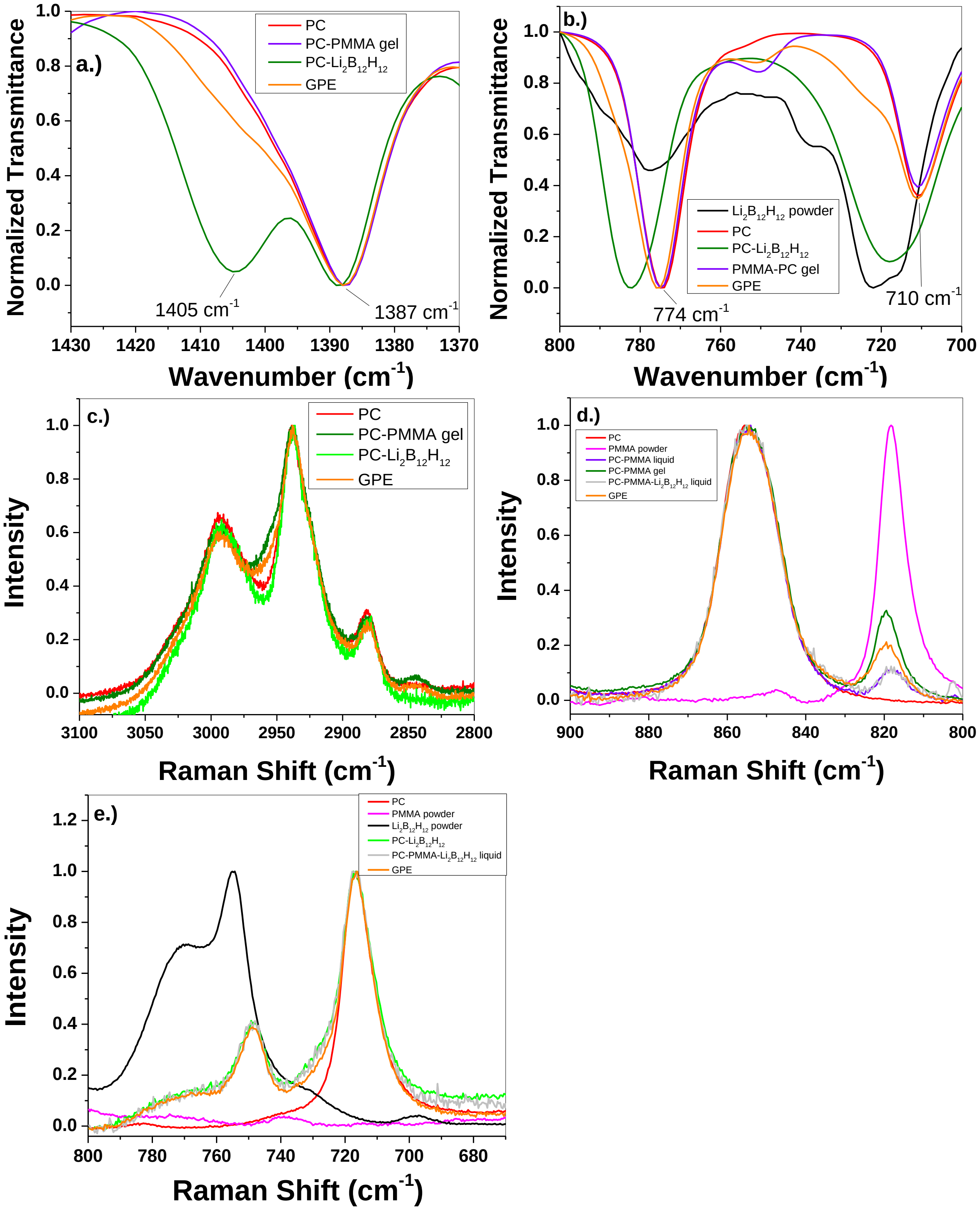

Figure S1 a-e. FTIR (a and b) showing a PC-$Li_2B_{12}H_{12}$ paste relative to the other components to highlight the shift of the –C-H_2 scissor mode of PC (a) and of the –C-H_2 rocking modes of PC (b). Raman spectra (c-e) of the GPE and its components at various stages of the synthesis.

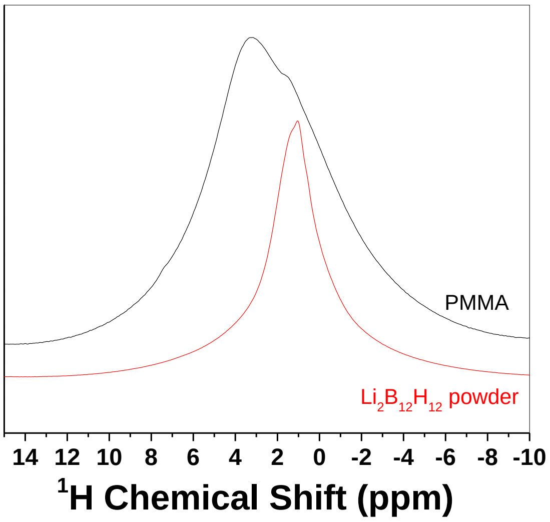

Figure S2. ^{1}H NMR spectrum of PMMA and $Li_2B_{12}H_{12}$ powders.

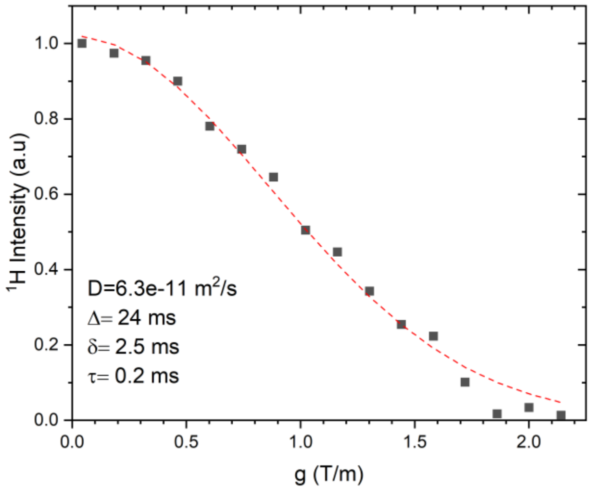

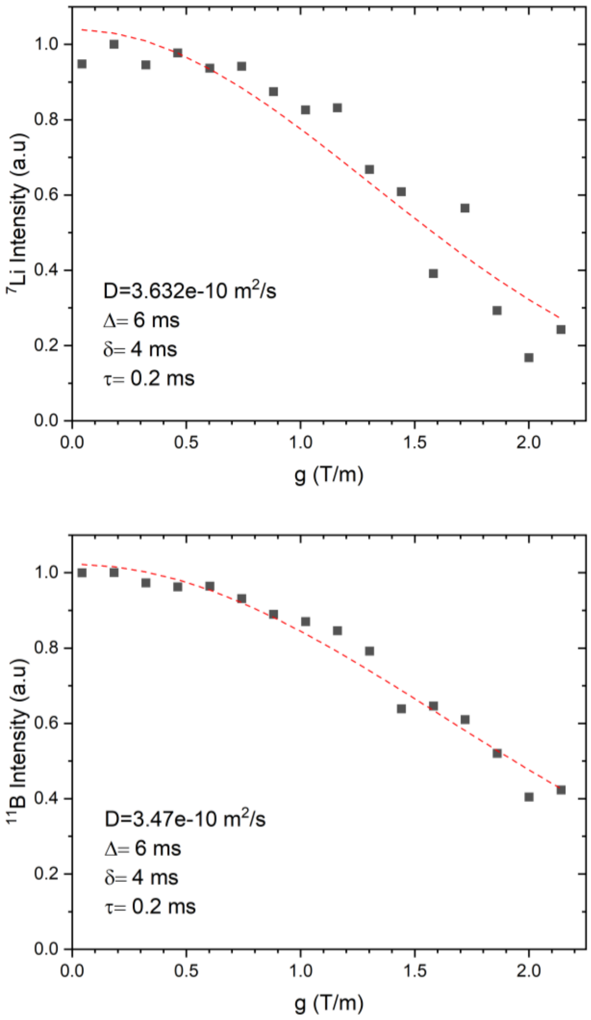

Figure S3. ^{1}H, ^{7}Li, and ^{11}B PFG-NMR attenuation data with fits of the Stejskal-Tanner equation for the GPE sample.

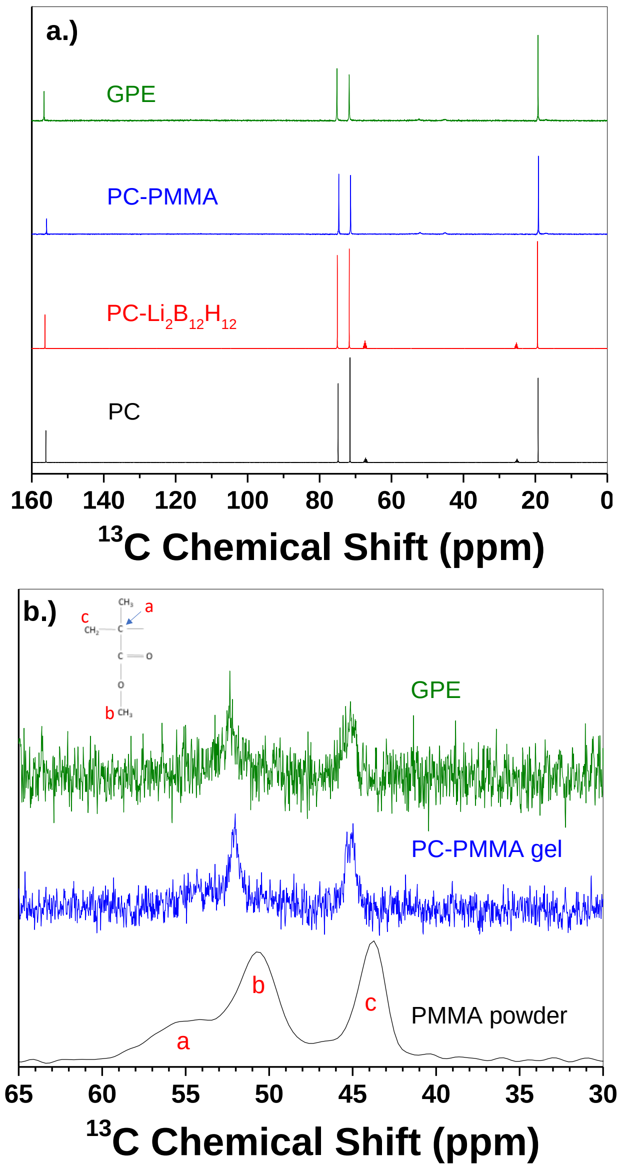

Figure S4. a.) Full ^{13}C NMR spectrum of the PMMA powder, PC-PMMA gel, and the GPE. b.) Zoomed in ^{13}C NMR spectrum highlighting the shift in the carbons from PMMA.

Table S1. Line fitting data for the multinuclear NMR experiments to show the percent contribution of the peak at each chemical shift to the spectrum.

**^{11}B GPE**		**^{7}Li GPE**		**^{13}C GPE**		**^{13}C PC/PMMA**	
**Chemical shift (ppm)**	**Relative Fraction %**	**Chemical Shift (ppm)**	**Relative Fraction %**	**Chemical Shift (ppm)**	**Relative Fraction %**	**Chemical Shift (ppm)**	**Relative Fraction %**
**-28.92**	9.23	-0.71	48.67	17.05	4.14	17.05	5.36
**-16.76**	2.85	-0.64	51.33	19.17	25.26	19.27	23.45
**-15.77**	23.56			45.13	4.61	45.18	4.31
**-15.42**	61.26			52.11	6.86	52.39	7.79
**-0.92**	3.09			71.39	26.75	71.77	24.15
				74.66	24.49	75.18	23.87
				155.9	4.41	156.59	8.63
				178.41	3.48	178.74	2.45

Table S2. ^{1}H NMR shifts predicted by DFT calculations

**^{1}H NMR**	**PC**	**Li-PC**	**4Li-PC**	**PC-$B_{12}H_{12}^{-2}$**
**Methyl group protons (11,12,7)**	1.39 ppm	1.85 ppm	1.57 ppm	1.76 ppm
**1**	4.30 ppm	5.07 ppm	4.58 ppm	5.82 ppm
**4**	4.62 ppm	5.46 ppm	4.93 ppm	5.22 ppm
**13**	3.75 ppm	4.49 ppm	4.01 ppm	4.06 ppm

Table S3. ^{13}C NMR Shifts predicted by DFT calculations

**^{13}C NMR**	**PC**	**Li-PC**	**4Li-PC**	**PC-$B_{12}H_{12}^{-2}$**
**6**	159.57 ppm	169.90 ppm	163.94 ppm	165.12 ppm
**5**	78.41 ppm	88.72 ppm	82.20 ppm	85.74 ppm
**2**	74.66 ppm	80.16 ppm	76.57 ppm	79.88 ppm
**9**	20.16 ppm	20.64 ppm	20.02 ppm	22.54 ppm

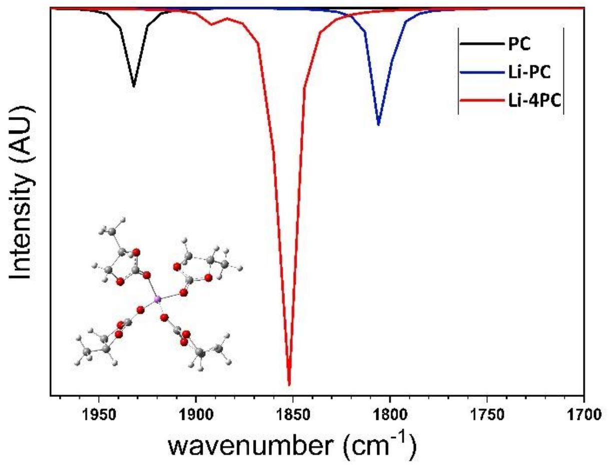

Figure S5. Vibrational frequency predictions for the PC carbonyl symmetric stretching mode (black) and coordination to lithium by one PC molecule (Blue) and coordination of 4 PC molecules with one lithium cation (red).

Table S4. Vibrational frequency predictions from DFT calculations demonstrating trend of frequency shift for PC vibrational modes in the presence of coordinated species

	**PC**	**Li-PC**	**4Li-PC**	**PC-$B_{12}H_{12}^{-2}$**
**C – H_2 rocking**	718 cm^{-1}	775 cm^{-1}	732 cm^{-1}	778 cm^{-1}
**C – H_2 rocking**	762 cm^{-1}	785 cm^{-1}	772 cm^{-1}	859 cm^{-1}
**C – H_2 scissor**	1382 cm^{-1}	1403 cm^{-1}	1395 cm^{-1}	1389 cm^{-1}
**C=O symmetric stretch**	1932 cm^{-1}	1804 cm^{-1}	1853 cm^{-1}	1876 cm^{-1}

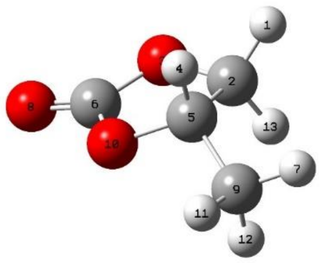

Figure S6. Structure of PC with labeled carbons and protons.

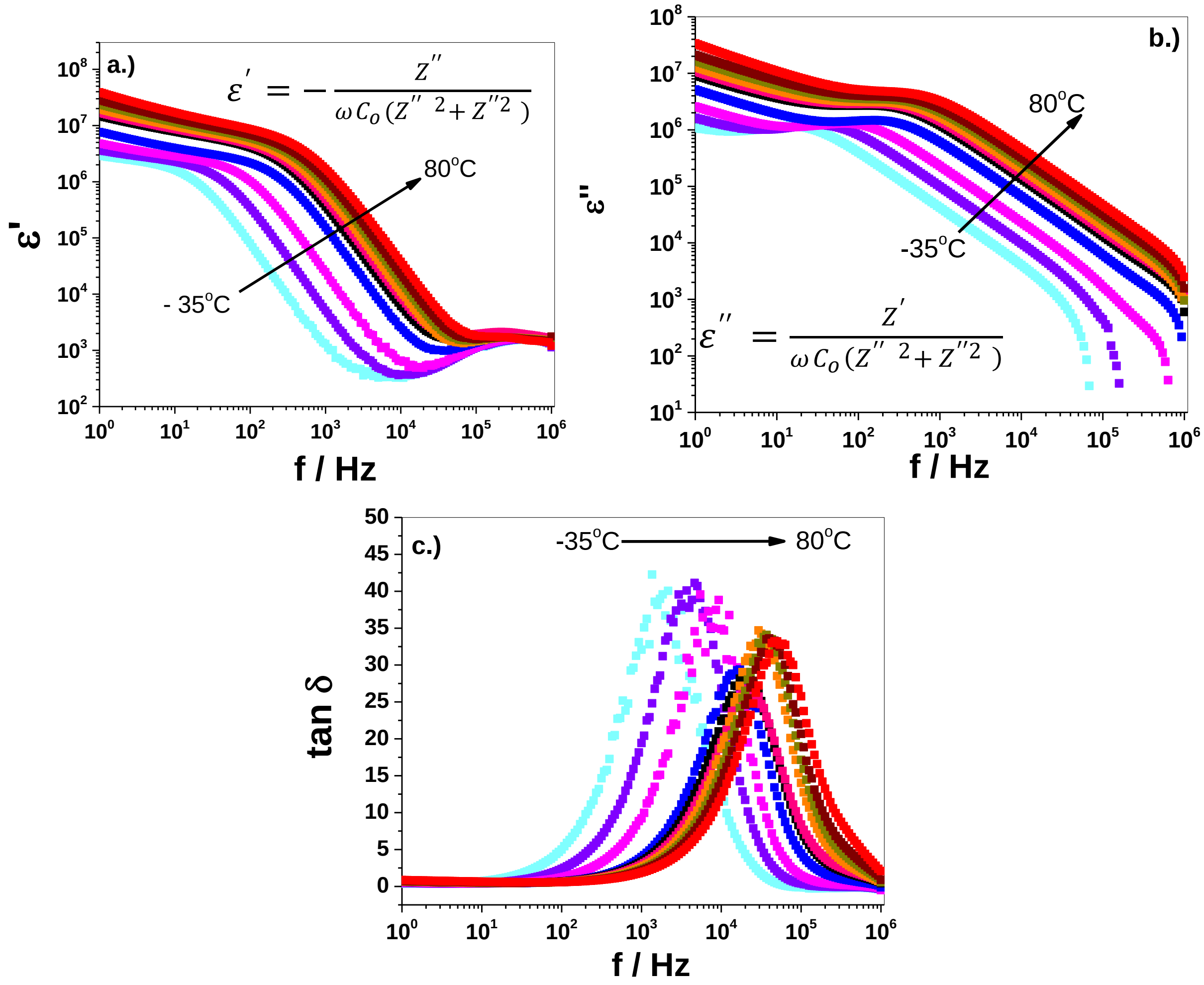

$$\varepsilon' = -\frac{Z''}{\omega C_o (Z''^{\,2} + Z''^{2})}$$

$$\varepsilon'' = \frac{Z'}{\omega C_o (Z''^{\,2} + Z''^{2})}$$

Figure S7. a.) Dielectric constant (ε'), b.) Dielectric loss (ε''), and c.) loss tangent (tan δ) for the GPE as a function of temperature. The corresponding equations utilized to calculate the (ε') and (ε'') from the EIS data are shown in the corresponding figures. In these equations, Z′ and Z″ are the real and imaginary parts of the dielectric constants, $C_o = \varepsilon_o A/t$, ε_o is the permittivity of free space (8.854 x 10^{-14} F cm^{-1}) and ω = 2πf, with f as the frequency.

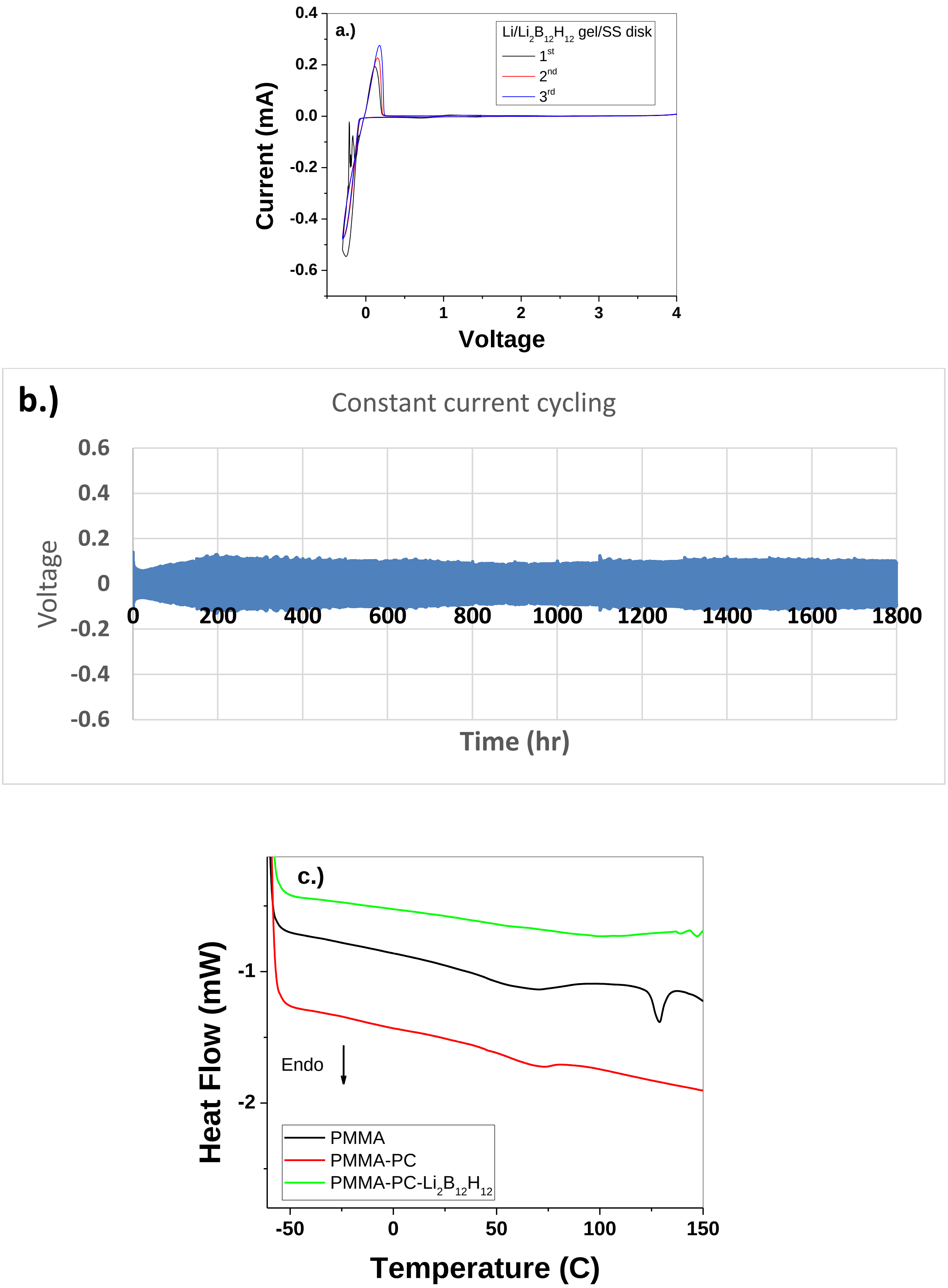

Figure S8. a.) Cyclic voltammogram of the Li/GPE/SS gel at a scan rate of 0.1 mV/s., b.) Full constant current cycling data performed on the GPE at a current density of 0.25 mA/cm^2. During the pandemic, there was limited lab access and greater than normal temperature fluctuations occurring during the course of this particular experiment due to maintenance

staffing issues. This variable temperature led to the periodic oscillations observed in portions of the data set with most of them occurring in 24 hour cycles. Additionally, due to the limited access to the facility, the cells often sat for days before the cycling was continued leading to a relaxation of the system further resulting in other oscillations for certain cycles. c.) DSC showing the PMMA, PC-PMMA gel, and the GPE. The PMMA shows a clear T_g at ~130 °C while the PC-PMMA and GPE do not show a clear T_g. This indicates the fluid and dynamic nature of the PMMA in the GPE leading to the enhanced Li^+ transport.

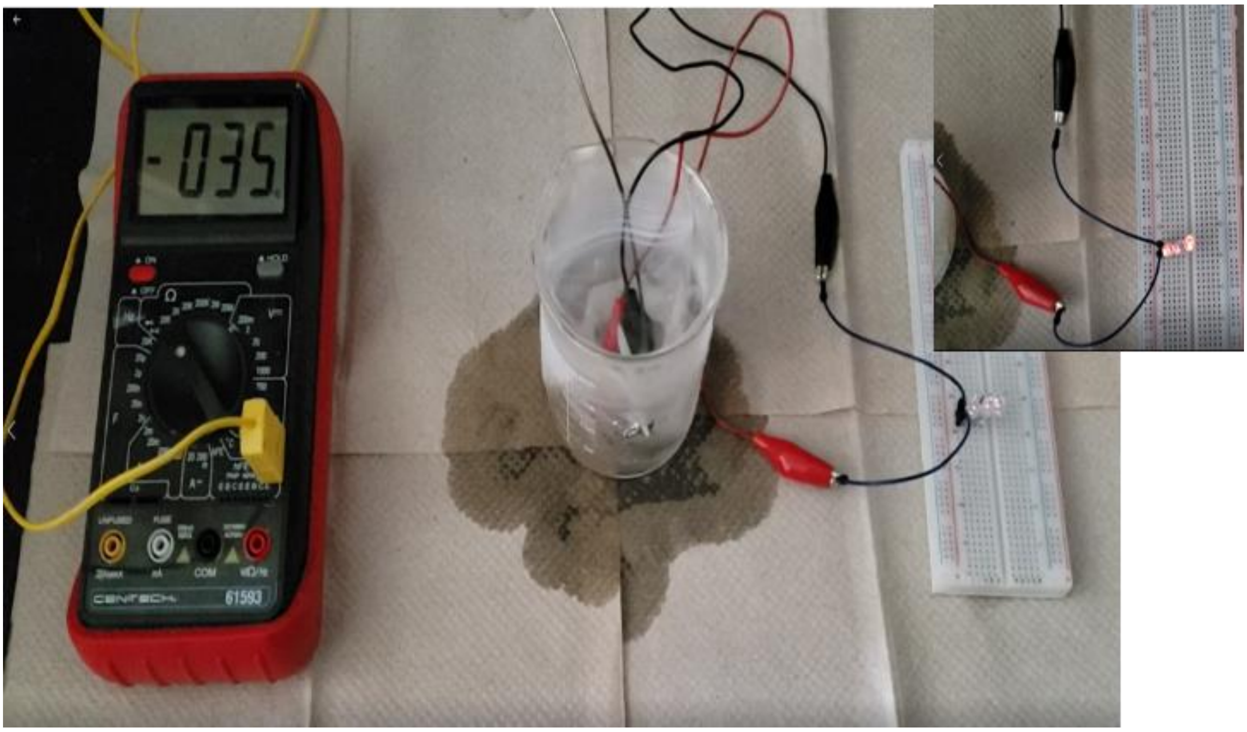

Figure S9. Image take from the video demonstrating the TiS_2/GPE/Li cell powering three LEDs at when submerged in a dry ice methanol bath at -35 °C after equilibration for 20 minutes (See Supporting Information video). Inset in the top right corner shows an overhead view of the directional red LEDs lit by the cell at -35 °C.

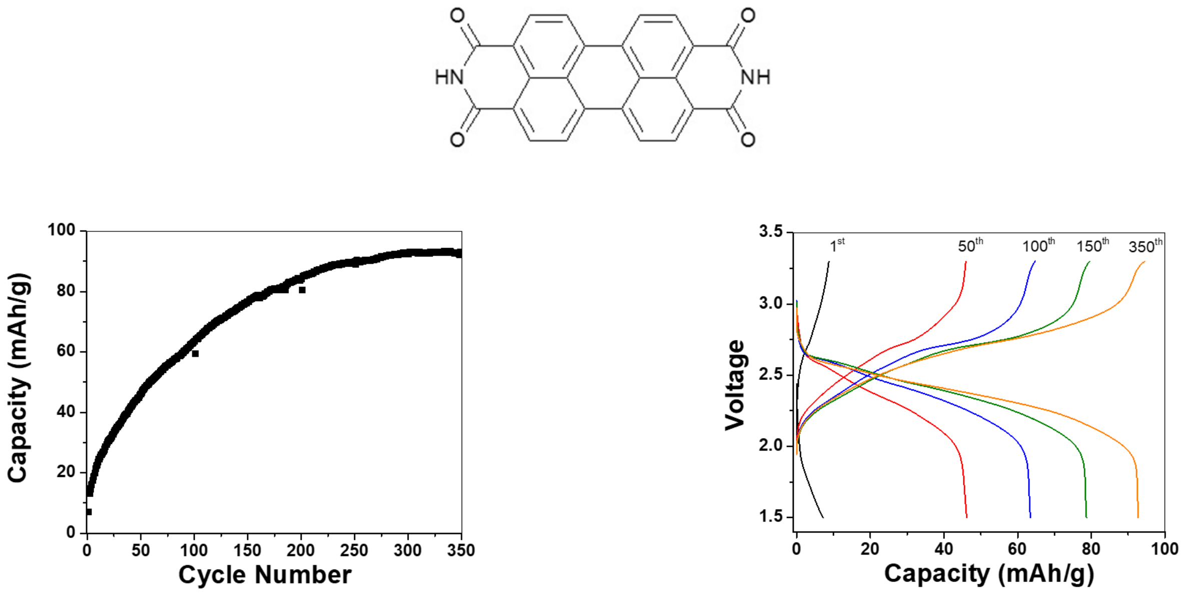

Figure S10. Structure of PTCDI, cycle performance, and charge/discharge profiles of the cathode as a function of cycle number.

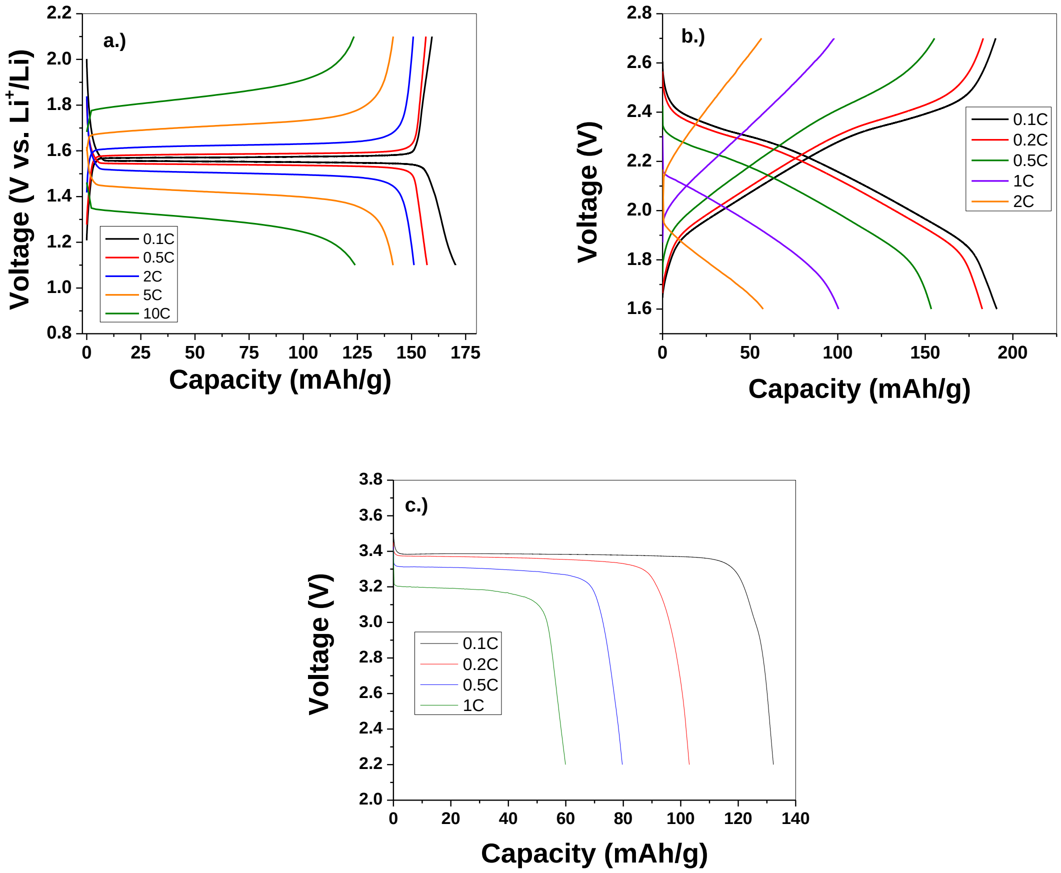

Figure S11. Galvanostatic cycling profiles for a.) LTO/GPE/Li, b.) TiS_2/GPE/Li, and c.) LFP/GPE/Li cell at different cycling rates. Based on Theoretical capacity of 175 mAh/g for LFP.
